# Early warning systems in obstetrics: A systematic literature review

**DOI:** 10.1371/journal.pone.0217864

**Published:** 2019-05-31

**Authors:** Aminu Umar, Charles A. Ameh, Francis Muriithi, Matthews Mathai

**Affiliations:** 1 Centre for Maternal and Newborn Health, Liverpool School of Tropical Medicine, Liverpool, United Kingdom; 2 Blackpool Teaching Hospitals National Health Service Foundation Trust, Blackpool, United Kingdom; University of Notre Dame Australia, AUSTRALIA

## Abstract

**Introduction:**

Several versions of Early Warning Systems (EWS) are used in obstetrics to detect and treat early clinical deterioration to avert morbidity and mortality. EWS can potentially be useful to improve the quality of care and reduce the risk of maternal mortality in resource-limited settings. We conducted a systematic literature review of published obstetric early warning systems, define their predictive accuracy for morbidity and mortality, and their effectiveness in triggering corrective actions and improving health outcomes.

**Methods:**

We systematically searched for primary research articles on obstetric EWS published in peer-reviewed journals between January 1997 and March 2018 in Medline, CINAHL, SCOPUS, Science Direct, and Science Citation Index. We also searched reference lists of relevant articles and websites of professional societies. We included studies that assessed the predictive accuracy of EWS to detect clinical deterioration, or/and their effectiveness in improving clinical outcomes in obstetric inpatients. We excluded studies with a paediatric or non-obstetric adult population. Cross-sectional and qualitative studies were also excluded. We performed a narrative synthesis since the outcomes reported were heterogeneous.

**Results:**

A total of 381 papers were identified, 17 of which met the inclusion criteria. Eleven of the included studies evaluated the predictive accuracy of EWS for obstetric morbidity and mortality, 5 studies assessed the effectiveness of EWS in improving clinical outcomes, while one study addressed both. Sixteen published EWS versions were reviewed, 14 of which included five basic clinical observations (pulse rate, respiratory rate, temperature, blood pressure, and consciousness level). The obstetric EWS identified had very high median (inter-quartile range) sensitivity—89% (72% to 97%) and specificity—85% (67% to 98%) but low median (inter-quartile range) positive predictive values—41% (25% to 74%) for predicting morbidity or ICU admission. Obstetric EWS had a very high accuracy in predicting death (AUROC >0.80) among critically ill obstetric patients. Obstetric EWS improves the frequency of routine vital sign observation, reduces the interval between the recording of specifically defined abnormal clinical observations and corrective clinical actions, and can potentially reduce the severity of obstetric morbidity.

**Conclusion:**

Obstetric EWS are effective in predicting severe morbidity (in general obstetric population) and mortality (in critically ill obstetric patients). EWS can contribute to improved quality of care, prevent progressive obstetric morbidity and improve health outcomes. There is limited evidence of the effectiveness of EWS in reducing maternal death across all settings. Clinical parameters in most obstetric EWS versions are routinely collected in resource-limited settings, therefore implementing EWS may be feasible in such settings.

## Introduction

The World Health Organization (WHO) estimated 303, 000 maternal deaths globally in 2015 at the end of the Millennium Development Goals era [1]. Over 99% of these deaths occurred in low-income settings [1]. It is also estimated that there were 27 million episodes of direct obstetric complications annually that contribute to long-term pregnancy and childbirth complications [2]. Good quality care including timely identification and management of obstetric complications can contribute to reducing the burden of maternal deaths and associated long-term complications [2].

**Fig 1:**
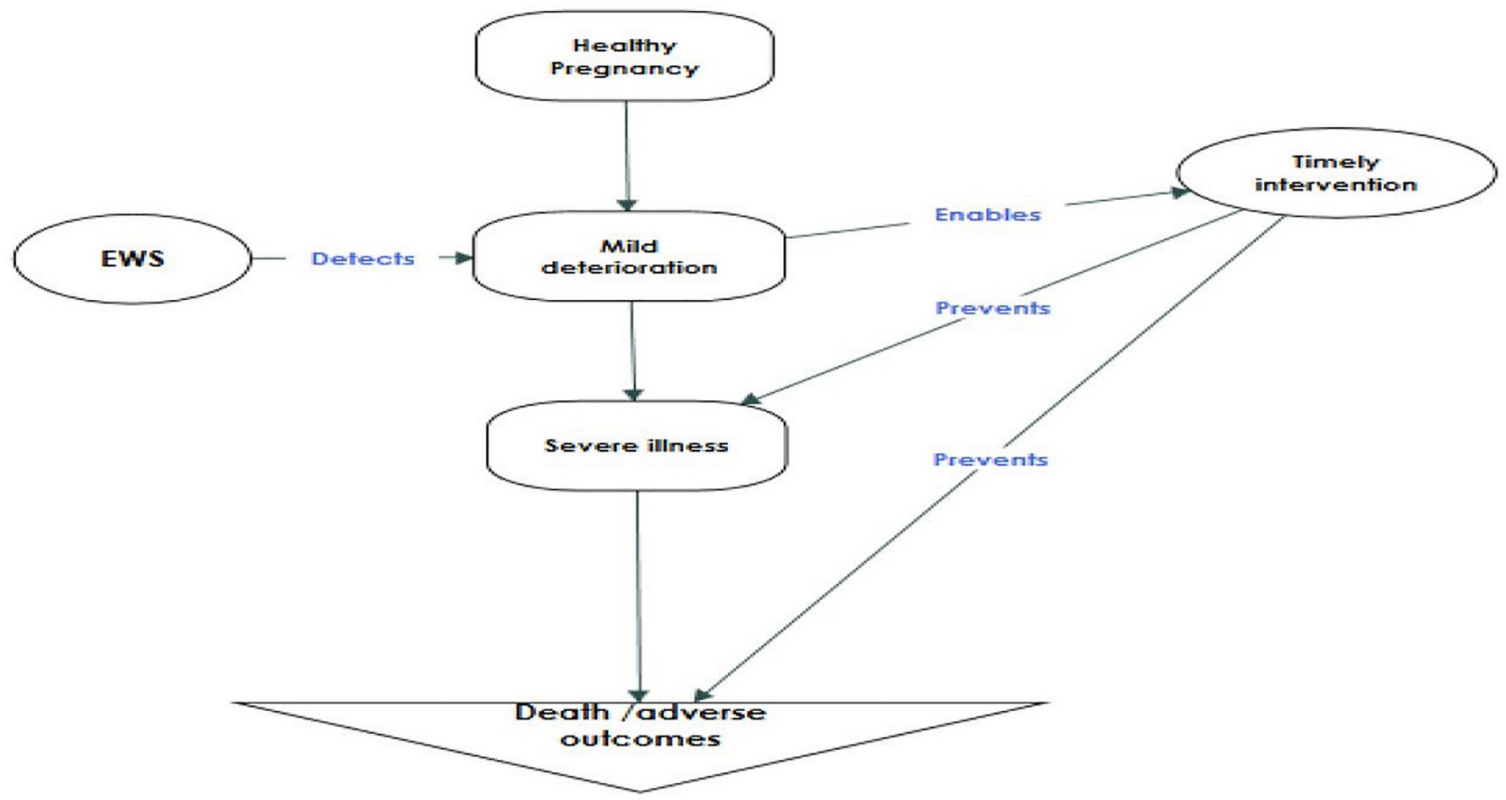
Hypothesis of the EWS intervention.

Early Warning Systems comprise clinical observation charts and algorithms for triggering corrective action to improve clinical outcomes. Early warning systems have been used in non-obstetric specialties since 1997 [3]. EWS combine clinical observations such as vital signs, clinical examination findings and laboratory tests to identify a pattern that is consistent with an increased risk of clinical deterioration. A trigger is defined as a single markedly abnormal observation or a combination of mildly abnormal observations. When a trigger is observed, it is expected that actions by the care team using a predefined protocol/algorithm will significantly reduce the risk of an adverse outcome[4]. Physiological clinical observations such as vital signs are different in pregnant women compared to non-pregnant women as are abnormal thresholds [5]. Modified early warning systems for the obstetric population have been advocated because they enable early detection of clinical deterioration, presenting an opportunity for timely actions to improve clinical outcome (Fig 1) [6].

The Saving Mothers’ Lives report of the United Kingdom’s Confidential Enquiry into Maternal Deaths (CEMDs) 2005 strongly recommended the adoption of EWS modified for the obstetric population [6]. Since then, EWS has been widely adopted for use in hospital maternities internationally. A survey of 130 UK hospital anaesthetists in 2014 identified different versions of obstetric EWS (varying number of clinical observations and pathophysiological thresholds to trigger clinical action); however, none of these was considered as the gold standard [7].

A systematic review of the effectiveness of obstetric EWS by Betesh et al. (2013) reported no direct evidence of improved clinical outcomes based on the two included observational studies with uncertain outcome measures [4,8,9]. Since that review, several obstetric EWS studies have been conducted, assessing the predictive accuracy of obstetric EWS for adverse outcomes, and their effectiveness in improving clinical outcomes. To the best of our knowledge, there has not been an up-to-date synthesis of evidence on the overall usefulness of EWS in obstetric practice.

The objectives of this systematic review were (1) to synthesise the evidence on effectiveness of obstetric EWS as screening tools for morbidity and mortality (predictive accuracy) and (2) to determine the effectiveness of the EWS trigger systems in improving clinical outcomes, and to explore the feasibility of their implementation in low resource settings.

## Methods

### Study design

Systematic review methodology was adopted to achieve the study objectives based on the principles and methods provided by the York University’s Centre for Reviews and Dissemination guideline. [10] The review findings were reported according to the Preferred Reporting Items for Systematic Reviews and Meta-Analyses (PRISMA) statement [11].

### Criteria for study selection

Study designs including prospective and retrospective longitudinal, case-control, cohort, quasi-experimental, step-wedge and randomized controlled trials were included if they possessed the following characteristics:

#### Participants

Pregnant women in labour, sick pregnant women of any gestational age and women who had recently given birth (within 6 weeks of delivery) admitted to hospital units including intensive care and high dependency units.

#### Intervention

Use of an obstetric EWS, including both paper-based and electronic monitoring systems.

#### Comparisons

Use of a non-obstetric EWS on an obstetric unit, usual care practice with no use of any EWS.

#### Outcome measures

Clinical outcomes: Maternal death, non-severe maternal morbidity, potentially life-threatening conditions, maternal near miss, intensive care unit admission. Trigger system: the need for a specialist review, referral for a higher level of care, the interval between a trigger and corrective clinical action.

### Search strategy

A preliminary search was conducted for existing reviews in the Cochrane central register, the three databases of the Centre for Reviews and Dissemination (Database of Abstract of Reviews of Effectiveness, Health Technology Assessment Database, and the NHS Economic Evaluation Database), Turning Research Into Practice (TRIP) Database and for ongoing reviews in PROSPERO.

We conducted a primary search of Medline, CINAHL, Scopus, Science Direct and Science Citation Index databases. We used search strategies that comprised a combination of text words and synonyms related to the intervention and outcomes of interest; search terms Appendix A in S1 Table. A systematic review expert at the Liverpool School of Tropical Medicine reviewed the search strategy that was subsequently piloted before application on the relevant databases. We also searched reference lists of identified articles and professional society websites including World Health Organization (WHO), Royal College of Obstetricians and Gynaecologists (RCOG), American College of Obstetricians and Gynaecologists (ACOG), Centre for Disease Control (CDC), Royal Australian and New Zealand College of Obstetricians and Gynaecologists (RANZCOG) and South African Society of Obstetricians and Gynaecologists (SASOG) for relevant publications. All relevant studies published between January 1997 to March 2018 in any language were included.

### Study selection

Two reviewers (AU and FM) independently screened all potentially relevant titles for the eligibility criteria. Publications were selected in two phases: first by reviewing titles and subsequently by a full-text review. Differences in judgment were resolved through consensus in consultation with CA and MM. All studies that met the inclusion criteria were included. Authors of conference abstracts were contacted by email for full texts: where these were not available, abstracts were excluded.

Studies were also excluded if they were: a) Conducted on a paediatric or non-obstetric adult population, b) Of qualitative methodological designs, c) Commentaries, editorials or letters.

### Data extraction

Data related to study title, author, design, setting, population, description of intervention used, outcomes and summary of findings of included studies were abstracted into a Microsoft Excel data abstraction sheet that was cross-checked and ratified by two reviewers.

### Data analysis

A structured narrative synthesis of included studies was conducted using the European Social Research Council guidance on the conduct of narrative synthesis in systematic reviews [12]. Included studies were tabulated by study objective and or study population to produce a clear descriptive summary. Relationships were explored within and between included studies; themes and sub-themes were identified and organized to fit the review’s objectives. The evidence was synthesized to provide a meaningful narrative. To determine the effectiveness of EWS as a screening tool for adverse obstetric outcomes, sensitivity, specificity, negative and positive predictive values, and Area Under Receiver Operating Characteristic Curve (AUROC) was analysed.

### Quality assessment

Based on one of the objectives of this systematic review—to synthesize evidence on the diagnostic accuracy of obstetric EWS- the QUADAS-2 (Quality Assessment of Diagnostic Accuracy Studies) tool was used for assessing the quality of included studies [13]. QUADAS-2 defines quality in diagnostic accuracy studies as the degree to which the estimate of diagnostic accuracy avoids the risk of bias, and the extent to which included studies are applicable to the review’s research questions. The included studies were assessed for risk of bias across four domains: patient selection, index test, reference standard and flow of participants [13]. Studies were also assessed for concerns about applicability across the first three domains. In accordance with the QUADAS-2 guidelines, the suggested signalling questions and scoring guideline were tailored to fit our review; Quality assessment tool Appendix B1 in S1 Table. We drew conclusions on the overall quality of included studies based on the frequency of low/high level of bias and low/high/unclear concerns about applicability in the four domains. We concluded that a study was of good quality when it had low-risk bias or concern about applicability in all four domains. A study was of moderate quality if it had no more than one unclear domain and no high risk of bias or concern about applicability. A study was of poor quality if it has more than one unclear domain or any high risk of bias/concern about applicability in the four domains.

The systematic review was registered in the International Prospective Register of Systematic Reviews (PROSPERO-CRD42017077504).

## Results

**Fig 2:**
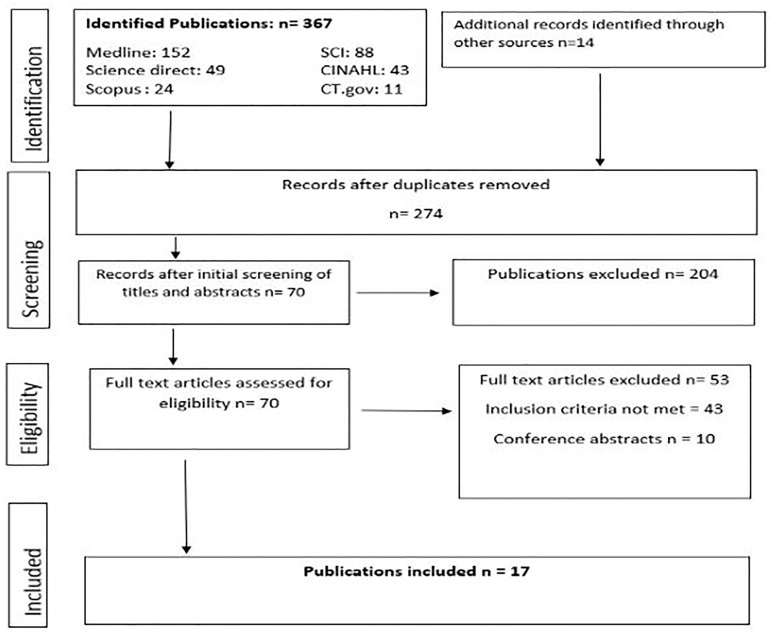
PRISMA diagram summarizing study selection process. Most of the studies that assessed the effectiveness of EWS in improving clinical outcomes were of quasi-experimental design.

Our search identified 381 papers (Medline = 152, Scopus = 24, CINAHL = 43, Science Citation Index = 88, Science Direct = 49, Clinical trials. gov = 11 and other sources = 14). Ten publications were available only as conference abstracts; authors of six of these abstracts confirmed unavailability of full texts. Seventeen papers met the inclusion criteria and were included in the review Fig 2, S2 Table. All studies that assessed the predictive accuracy of EWS for adverse obstetric outcomes were observational studies.

### Characteristics of included studies

Included studies fell into two thematic categories: studies that investigated the predictive accuracy of EWS for adverse obstetric outcomes (validation studies) and those that investigated the effectiveness of EWS in improving measured outcomes (clinical outcomes and measures of the effectiveness of the EWS trigger mechanism). The study characteristics, including design, participants, intervention, outcome measures and key findings of these studies are presented in Table 1. All reviewed studies were published between 2010 and 2017.

**Table 1:** Summary of included studies.

**Theme 1**	Studies that tested the predictive accuracy of EWS on adverse obstetric outcomes				
**Publication**	**Design**	**Study Population**	**EWS type**	**Outcomes**	**Findings**
Lappen RJ et al., 2010	Retrospective cohort	Women with chorioamnionitis (n = 913)	MEWS and SIRS	Severe sepsis, ICU transfer, death	Both performed poorly (MEWS PPV = 5.4%, SIRS Specificity = 17.6%)
Von-Dadelszen P et al., 2011	Prospective Multicentre cohort study	Women admitted with pre-eclampsia or who developed pre-eclampsia in hospital (n = 1935)	fullPIERS model	Death, 1 or more serious CNS, cardiorespiratory, hepatic, renal, or haematological morbidity.	Predicted adverse maternal outcomes with AUROC of 0.88 (95% CI 0.84–0.92)
Singh S et al., 2012	Prospective observational study	Obstetric admissions from 20 weeks through to 6 weeks post-partum (n = 676)	CEMACH MEOWS	Outcome at 30 days- Morbidity based on consensus, death, ICU admission, discharged alive.	30% (200) triggered, 13% (86) had morbidity. Sensitivity 89% (95% CI 81–95%), Specificity 79% (95% CI 76–82%) PPV 39% (95% CI32-46%), NPV 98% (95% CI 96–99%)
Carle C et al., 2013	Retrospective analysis of secondary data	Obstetric admissions (n = 4440) to ICU	ICNARC obstetric EWS	Death	AUROC Statistical EWS = 0.99 Clinical EWS = 0.96
Payne et al., 2014	Prospective Multicentre cohort study	Women (n = 2081) with any hypertensive disorder of pregnancy admitted to a participating centre.	miniPIERS model	Death, 1 or more serious CNS, cardiorespiratory, hepatic, renal, or haematological morbidity	Predicted adverse maternal outcomes with AUROC of 0.77 (95% CI 0.74–0.80)
Edwards ES et al., 2015	Retrospective cohort	Women with chorioamnionitis (n = 913)	Six published MOEWS charts	Severe sepsis, death	AUROCs: A = 0.65 B = 0.52 C = 0.52 D = 0.72 E = 0.68 F = 0.65
Singh A et al., 2016	Prospective observational study	Women in labour beyond 28 weeks gestation, up to 6 weeks postpartum (n = 1065)	CEMACH MEOWS	Morbidity based on consensus	Sensitivity 86.4% Specificity 85.2% PPV 53.9% NPV 96.9%
Hedriana HL et al., 2016	Retrospective case-control study	Cases; Obstetric admissions to ICU (n = 50), Controls; SVD (n = 50)	MEWT	ICU admission	Sensitivity 72% (95% CI 57–83%) Specificity 96% (95% CI 85–99%) PPV 95% (95% CI 81–99%) NPV 77% (95% CI 65–87%)
Shields E L et al. 2016	Quasi-experimental	Obstetric admissions in 6 hospitals n = 11399	MEWT	ICU admission	Sensitivity 97% Specificity 99% PPV 12% NPV 99%
Ryan HM et al., 2017	Retrospective case-control study	Cases; 46 obstetric admissions to ICU, Controls; 138 admissions no critical care	CEMACH MEOWS	ICU admission for longer than 24 hours	Sensitivity 96% Specificity 54% PPV 41% NPV 97%
Paternina-Caicedo et al., 2017	Retrospective cohort study	Pregnant and postpartum women (up to 42 days) admitted into the ICU (n = 702) due to direct and indirect obstetric causes.	ICNARC obstetric EWS	Death	AUROC 0.84 (AUROC of 0.87 in direct and 0.77 in indirect obstetric admissions)
Nathan HL et al., 2017	Prospective cohort	Women with preeclampsia at admission (n = 1547)	CRADLE Vital Signs alert EWS	Kidney injury, MgSO4 use, and ICU admission, death	Trigger predicted an increased risk of Kidney injury (OR 1.74), MgSO4 use (OR 3.4) and ICU admission (OR 1.5)
**Theme 2**	Studies testing the effectiveness of EWS in improving measured outcomes in an obstetric population				
**Publication**	**Design**	**Participants**	**EWS**	**Outcomes**	**Findings**
Austin DM et al., 2013	Mixed retrospective (before) and prospective (after) design	Retrospective (n = 42) and prospective (n = 71) obstetric admissions	EWS	Severity of morbidity	MDT review determined that EWS might have reduced severity of morbidity, by 7.6%
Maguire PJ et al., 2015	Mixed retrospective (before) and prospective (after) design	Obstetric patients with bacteraemia before (n = 61) and after (n = 20) IMEWS	IMEWS	Vital signs recording and trigger/antibiotic time lag	Improvement in RR recording (p<0.05) and reduction in time between trigger and antibiotics (p>0.05)
Maguire PJ, 2016	Retrospective observational study	Women monitored with IMEWS (n = 80) and other methods (n = 87) before ICU admission	IMEWS	ICU Admission	IMEWS contributed to early recognition of critical illness (in 73.8% of participants, n = 80) but cannot replace clinical judgment
Shields E L et al. 2016	Quasi-experimental	Obstetric admissions in 6 hospitals n = 11399	MEWT	CDC defined maternal morbidity, ICU admission	Reduction in morbidity (p = 0.01) and ICU admission (p = 0.8)
Sheikh S et al., 2017	Before after Quasi-experimental	Women who had CS before (n = 100) and after (n = 100) implementation of NEWS	NEWS	Need for specialist review, ICU admission, referral due to post-op complications, death	No statistically significant difference
Merriel A et al., 2017	Before after Quasi-experimental	Women undergoing CS before (n = 79) and after (n = 85) implementation	MEOWS	Pre-operative stabilization, action taken due to trigger	Significant improvement in the two outcomes (p<0.05). pre-op stabilization improved after MEOWS: odds ratio 2.78, 95% CI, 1.39–5.54. Improved care triggered in 68% of patients after EWS compared to 4% before (p<0.001)

**AUROC**: Area Under Receiver Operating Characteristic Curve, **EWS**: Early Warning Systems, **ICU**: Intensive Care Unit, **IMEWS**: Irish Maternity Early Warning System, **MEOWS**: Modified Early Obstetric Warning Systems, **MEWT**: Maternal Early Warning Triggers, **NEWS**: National Early Warning System, **NPV**: Negative Predictive Value, **PPV**: Positive Predictive Value

The studies were distributed across six high-income countries, 3 upper-middle-income countries (Colombia, South Africa, and Brazil), one lower-middle income (India) and two low-income countries (Zimbabwe, Uganda). There were two multi-country studies: one of these [14] was conducted in five high-income countries (Canada, Australia, New Zealand, and the UK), and the other [15] recruited participants from five low and middle-income countries, including India, Pakistan, Zimbabwe, Colombia, South Africa, and Brazil.

Twelve observational studies [4,5,9,15–23] assessed effectiveness of EWS in predicting obstetric morbidity and mortality (predictive accuracy); of these, seven [4,9,16,19,20,22,23] investigated accuracy of the tools in predicting adverse outcomes among all obstetric inpatients, of these two were prospective studies and four were retrospective studies. Five were validation studies that looked at specific obstetric outcomes associated with chorioamnionitis [5,21] and pre-eclampsia [14,15,17].

Four studies tested the effectiveness of EWS in reducing the prevalence of measured outcomes; the severity of morbidity [22,24], ICU admission [22,25,26] and pre-operative stabilisation [27]. Three studies reported on measures of EWS trigger effectiveness: referral rate for further care [26], change in frequency of vital sign monitoring [22], change in the interval between a trigger and corrective clinical action [27,28]. One study [22] assessed both the predictive accuracy of EWS and its effectiveness in improving clinical outcomes.

### Quality of included studies

**Fig 3:**
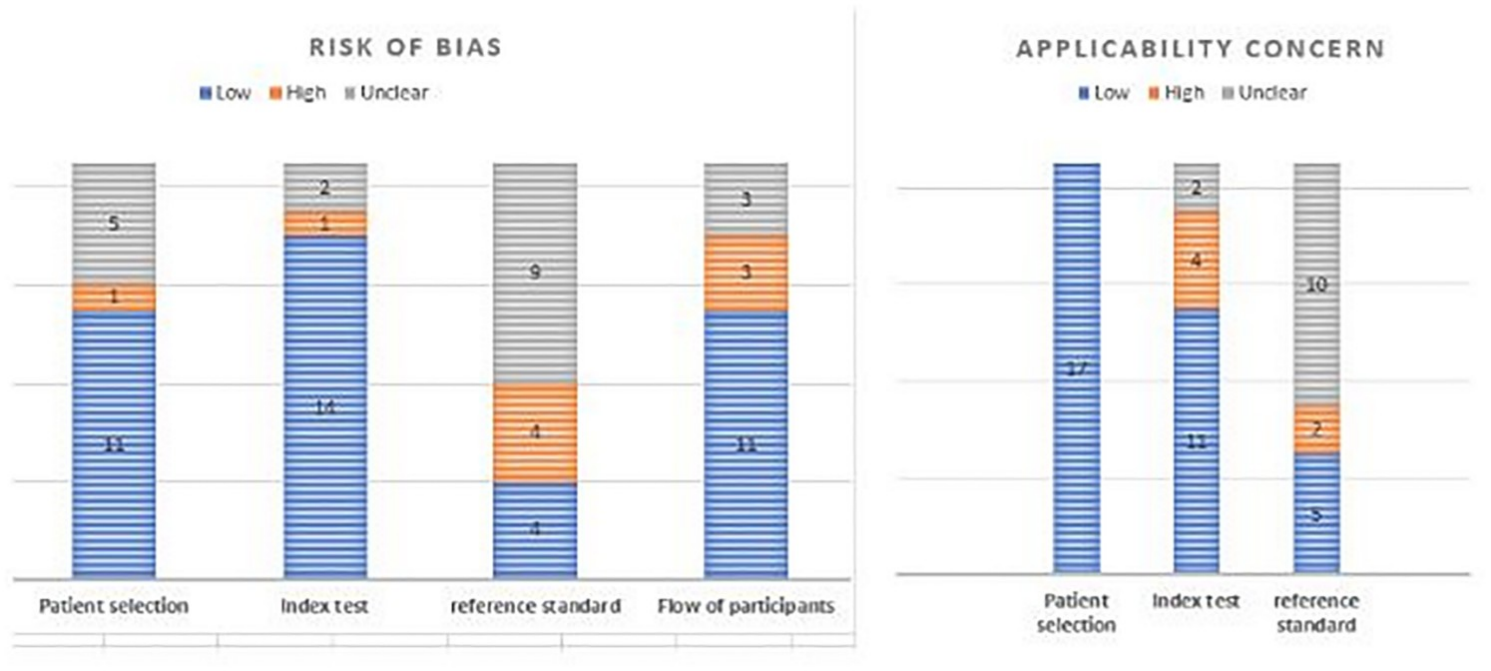
Quality assessment of included studies (n = 17).

Based on the QUADAS-2 tool, a summary of the quality of all included studies is presented in Fig 3, while a detailed assessment of the quality of each included studies (risk of bias and concern about applicability) is provided as Appendix C in S1 Table.

#### Risk of bias

The majority of included studies had a low risk of bias for patient selection (65%), index test (82%) and the flow of participants (65%) Fig 3. The most common source of high risk of bias was the absence of a reference standard (4 studies), followed closely by the flow of participants or attrition bias in (3 studies), Fig 3. Just over a half of the included studies (9, 52%) had an unclear risk of bias for a reference standard; this was commonest among studies that assessed the effectiveness of EWS on measured outcomes Appendix C in S1 Table.

#### Concern about applicability

There was a high concern about the applicability of EWS used in four studies to this review’s research questions: two studies used EWS that were not modified for obstetric populations. [5,26] The other two [14,15] used statistically derived EWS generated from multi-country prospective cohort studies designed for use in pre-eclampsia patients only. The resulting EWS included vital signs, laboratory investigations, and clinical signs, and may not be applicable to all obstetric patients or in low resource settings.

### Diagnostic accuracy of EWS

Seven of the 11 included studies that tested the effectiveness of EWS in predicting obstetric morbidity/mortality had death as a primary outcome Table 1.

#### Accuracy in predicting maternal death

Two studies were retrospective, of good or moderate quality (Appendix C in S1 Table) and were on all obstetric patients admitted to intensive care units. Both studies showed that the obstetric EWS had a very high accuracy in predicting death (AUROC >0.80) among critically ill obstetric patients [9,16].

Five studies on specific obstetric populations had a high risk of bias or concern for applicability Appendix B2 in S1 Table [5,15,17,18,21]. Three of these were large prospective studies that focused on women with pre-eclampsia and reported high sensitivity (>85%), specificity (>75%) and AUROC (>0.75) of EWS in predicting death. [15,17,18] Two retrospective studies with pregnancy-related sepsis only population, showed that EWS did not accurately predict death (PPV <10%, AUROC < 0.75) [5,21].

#### Accuracy in predicting morbidity and ICU admission

Five studies [5,17,20,22,23]reported the accuracy of obstetric EWS in predicting increasing severity of obstetric morbidity or ICU admission. General obstetric study populations were used in three of these studies. [20,22,23] Although these studies were of varying quality, they reported that EWS had a very high median (inter-quartile range) sensitivity of 89% (72% to 97%) and specificity of 85% (67% to 98%). The median (inter-quartile range) positive predictive values were low—41% (25% to 74%) Table 1.

Two retrospective studies reported very poor predictive accuracy (PPV less than 10% and AUROC of less than 0.75) of obstetric EWS for severe sepsis among cohorts of women with chorioamnionitis. [5,21] Table 1. However, the dataset used in the two studies had a high risk of attrition bias as more than 50% of the participants had incomplete vital signs records and were excluded from the analysis; Appendix C in S1 Table.

### The effectiveness of EWS in improving clinical outcomes

Six studies [22,24–27,29]assessed the effectiveness of EWS in improving outcomes in general obstetric populations. The outcome measures included clinical outcomes (morbidity, maternal death, and ICU admission) and trigger system measures (vital sign recording, the time lag between the trigger and corrective clinical action, preoperative stabilization, need for specialist review and referral rate) Table 2.

**Table 2:** Outcomes assessed by the EWS effectiveness studies (n = 6).

	Outcome measures						
Publication	Morbidity	ICU admission	Maternal Death	Vital sign recording	Time lag*	Preop stabilization**	Referral rate^#^
Austin DM et al., 2013	✓						
Maguire PJ et al., 2015				✓	✓		
Maguire PJ et al., 2016		✓					
Shields E L et al. 2016	✓	✓					
Sheikh S et al., 2017		✓	✓				✓
Merriel A et al., 2017						✓	

*Time lag: time interval between trigger and review.

**Preop stabilization: clinical actions taken to optimize patients undergoing a caesarean section.

^#^ Referral rate: rate of referral of sick patients to a higher level of care, including critical/intensive care

#### Maternal morbidity, death and ICU admission

Only one before and after study in patients who had had a caesarean section had maternal death as an outcome measure [26]. In that study, the two periods (pre and post-EWS implementation) compared were not equal, it was unclear if the sample was large enough to detect any difference in the outcome. However, there was a significant reduction in complications due to post-partum haemorrhage (PPH) after EWS introduction (Table 1). The reduction in PPH after the introduction of the EWS compared to before was attributed to early recognition and timely management.

In a large quasi-experimental study there was a significant reduction in CDC-defined severe and composite maternal morbidity (p<0.01) but not mortality in 6 intervention hospitals following EWS implementation, compared to 19 control hospitals. Also, there was no change in the ICU admission rate in the intervention and control hospitals [22].

There was a non-significant reduction in ICU admission and severity of obstetric morbidity after implementation of EWS in two before and after studies [24,29] Table 1.

#### Quality of patient care

One before-after study (before n = 61, after n = 20) reported an increase in the frequency of documentation of vital signs (specifically for respiratory rate) following the implementation of the Irish Maternity Early Warning System [25]. The authors also reported a statistically significant reduction in the time interval between EWS trigger and antibiotic administration for obstetric patients with bacteraemia.

Pre-operative stabilization of women undergoing caesarean section was reported to have significantly improved after implementation of EWS (Odds ratio 2.78, 95% CI, 1.39–5.54) [27]. Also, there were improvements in care triggered by abnormal EWS observations in 68% of patients after EWS implementation compared to only 4% before (p<0.001).

In another before-after study (before n = 100, after n = 100), Sheikh and colleagues [26] reported a non-statistically significant reduction in the need for specialist review. Similarly, there was a non-significant higher-level referral rate due to post-operative complications among women who had a caesarean section, after implementation of EWS.

### EWS parameters

Details of 16 versions of EWS were identified from the reviewed studies. The components of the EWS used in one study was not specified [26]. There were variations in parameters included among the reviewed EWS charts; however, 14 of the 16 charts had pulse rate, systolic blood pressure, and respiratory rate, 13 charts had temperature and 12 charts had diastolic blood pressure and conscious level. On average four in five charts identified in this review (mean, 82.5%; n = 16) had these 5 parameters.

## Discussion

Our systematic review did not identify any randomised controlled trials on EWS. It included 17 studies, mostly observational studies [11] and only two of all included studies were conducted in low-income countries. All studies that assessed the predictive accuracy of EWS for adverse obstetric outcomes were observational studies. Most of the studies that assessed the effectiveness of EWS in improving clinical outcomes were of quasi-experimental design.

For a screening tool to be of value, it should be safe to use, cost effective, accurate and acceptable to care, providers. The accuracy of an early warning chart to predict morbidity is indicated by the positive or negative predictive value (PPV/NPV). Both of these are dependent on the prevalence of the condition. While it is desirable that a screening test should have a high sensitivity and specificity, the probability of a positive result when the condition actually exists (PPV) or the probability of a negative result when the condition does not exist (NPV) is equally important. A screening tool for a condition of low prevalence, with high sensitivity and specificity, will likely have a low PPV and a high NPV. While for more common conditions, a screening test/tool with similar sensitivity and specificity will likely have a high PPV and a low NPV.

Early warning systems developed using a statistically derived model for obstetric population admitted to the critical care unit are accurate in predicting death (AUROC >0.80) [16]. In other general obstetric population, EWS haswas shown to be highly sensitive and specific in predicting morbidity and ICU admission, with comparatively low PPV (average of 41%). With a low probability that subjects with a positive screening test are truly at risk of deterioration (low PPV), there is the risk of unnecessary use of resources when protocols are triggered due to a ‘positive’ test. Similarly, EWS with low NPV may miss many women who are likely to deteriorate clinically by giving them a false resuarrance, and potentially resulting in catastrophic outcomes.

One low quality multicentre controlled trial reported that obstetric EWS significantly reduces CDC-defined maternal morbidity but not ICU admission rate [22]. A reduction in ICU admission will have been expected because the implementation of corrective measures may reduce the need for ICU admission however the management of women who are predicted to develop morbidity may be best in the ICU. Therefore ICU admission rate may not be a good outcome measure because the criteria for ICU admission may vary.

The low positive predictive values for severe morbidity and ICU admission (PPV 41%) means that approximately, only one in two cases with a positive screening test is truly at risk of deterioration. As pointed out by Friedman, [30] a warning system with a high false-positive rate, may potentially worsen clinical care, constitute a nuisance alarm and contribute to alarm fatigue.

However, the relatively low positive predictive value for obstetric morbidity and ICU admissions reported in this review is comparable to other non-obstetric aggregated and single parameter early warning systems [31,32]. Hence, as with these non-obstetric EWS, and as pointed out by Maguire and colleagues, [29] obstetric EWS needs to be used with, and do not substitute, clinical judgment in patient monitoring and care.

Based on one small sample low-quality quasi-experimental study, there is no evidence that EWS reduces maternal deaths [26].

There is some evidence from small sample size, low-quality studies that introduction of EWS improves the quality of care for obstetric patients. Specifically, EWS significantly improves the frequency of vital sign observation, and improves pre-caesarean section stabilization of patients [26,27]. Also Maguire et al. in a small sample before after study, reported that EWS reduces the time interval between abnormal vital signs and implementation of corrective clinical action but this was not statistically significant.[25]

There are some conflicting findings, particularly on the use of EWS in women with chorioamnionitis. Lappen et al.[5] and Edwards and colleagues [21] reported very poor performance for predicting sepsis in women with chorioamnionitis and argued that the EWS should not be used in this population. However, the findings were not surprising because information of vital signs was missing from records of 549 of the 913 women and was excluded from the analysis [21,28]. For this reason, we assessed the two studies as having a high risk of attrition bias; Appendix C in S1 Table.

Fourteen of the 16 EWS versions identified in this review included five parameters; the pulse rate, respiratory rate, temperature, blood pressure, and consciousness level. These parameters need simple patient monitoring devices that are readily accessible (BP machine, a thermometer, and a clock or timer) to measure. This finding suggests that EWS may be feasible to implement in low-resource settings where more sophisticated monitoring and diagnostic equipment may be unavailable [33]. However, evidence from a prospective cohort study identified the need for local validation and impact assessment of EWS tools before their adoption in resource-limited settings [34].

This systematic review provides more information than the previous systematic review in 2013 [8], on the predictive accuracy and effectiveness of obstetric EWS. Our results support the hypothesis that EWS may improve the quality of monitoring of obstetric patients, possibly resulting in improved reaction time by clinical staff to prevent further deterioration. These findings agree with outcome improvement reported with EWS in non-obstetric patients population [32].

To the best of our knowledge, this is the first systematic review to report predictive accuracy, and effectiveness on clinical outcomes of obstetric EWS. Other strengths of this review include adherence to the good practice of protocol registration and use of a robust tool for quality assessment in diagnostic accuracy studies. A limitation of our review is the lack of standardization of the defining criteria for outcomes. For instance, maternal morbidity was defined based on the CDC-criteria in the study by Shields et al., [22] while other studies [4,19,24] defined morbidity based on consensus among authors. We, therefore, identified a need to standardize outcomes in EWS effectiveness studies for clinical and research purposes. Most of the studies included were observational studies and only one of the studies that assessed the effectiveness of EWS in improving clinical outcomes had death as a primary outcome. More robust studies with large sample sizes are required to detect the effect of EWS on maternal deaths.

There were different versions of obstetric EWS across hospitals in keeping with lack of standardization as reported for non-obstetric systems. [32] This can result in a lack of familiarity with local systems when staff move between clinical areas and hospitals.

Finally, the 12 EWS validation studies revealed a strong association between high scores and adverse obstetric outcomes. However, only one study assessed the time interval between the EWS trigger across different parameters and intravenous antibiotic administration [29]. Robust studies, for example, cluster randomised controlled trials, with the interval between a trigger and corrective clinical action as an outcome measure, are needed.

## Conclusion

Obstetric EWS are highly sensitive and specific in predicting obstetric morbidity and ICU admission with relatively low, but comparatively acceptable PPV. This supports their utility as valuable bedside screening tools for morbidity among the general obstetric population. Early warning systems are highly accurate in predicting maternal death among critically ill obstetric patients, but there is limited evidence of their effectiveness in reducing maternal deaths. Obstetric EWS may improve the frequency of routine vital sign observation and may reduce the interval between patient deterioration and corrective clinical action. These can potentially improve the quality of care for pregnant/postpartum women and reduce the risk of adverse obstetric outcomes. Most obstetric EWS versions have basic clinical observations that can be routinely collected in resource-limited settings making them feasible for use in such settings. More robust studies are however needed to assess the effectiveness of obstetric EWS in reducing maternal deaths.

## Supporting information

S1 Table Appendix A-C (A-combination of serch terms, B1-quality assessment tool, B2-QUADA 2 scoring guideline, C-quality of individual studies). (PDF)

S2 Table PRISMA check list. (DOC)

## Abbreviations

AUROC: Area Under Receiver Operating Characteristic Curve
CDC: Centre for Disease Control
CEMACH: Confidential Enquiry into Maternal and Child Health
EWS: Early Warning Systems
ICU: Intensive Care Unit
IMEWS: Irish Maternity Early Warning System
MEOWS: Modified Early Obstetric Warning Systems
MEWT: Maternal Early Warning Triggers
NEWS: National Early Warning System
NPV: Negative Predictive Value
PPV: Positive Predictive Value
RANZCOG: Royal Australian and New Zealand College of Obstetricians and Gynaecologists
SASOG: South African Society of Obstetricians and Gynaecologists
RCOG: Royal College of Obstetricians and Gynaecologists
WHO: World Health Organization
