# Supplementary material for: Early warning systems in obstetrics: A systematic literature review
Source: PLoS One. 2019 May 31;14(5):e0217864. doi: 10.1371/journal.pone.0217864 (PMC6544303; doi:10.1371/journal.pone.0217864)
Supplement: S1 Table — (PDF) [file pone.0217864.s001.pdf]

Appendices

Appendix A: Combination of Search terms

Intervention terms	Outcome terms
“early warning”	“maternal mortality” (MeSH)
“early detection”	“maternal morbidity” (MeSH)
“track and trigger”	“obstet* complication”
“monitoring chart”	“obstet* haemorrhage”
“vital$ chart”	“matern* outcome”
EWS	“matern* sepsis”
MOEWS	“eclampsia”
MEOWS	“chorioamnionitis”

Appendix B1: Quality assessment tool

Domain	Signalling questions	Scoring
**Patient selection**<br>Risk of bias	Was a continuous or random sample of participants enrolled?	Yes<br>No<br>Unclear
	Did the study avoid unnecessary exclusions?	Yes<br>No<br>Unclear
Applicability concern	Are there concerns that the included participants do not match the review questions?	Yes<br>No<br>Unclear
**Index test**<br>Risk of bias	Were the reference range adjusted for physiological change in pregnancy?	Yes<br>No<br>Unclear
	Was the trigger threshold prespecified?	Yes

			No<br>Unclear
	Applicability concern	Are there concerns that the index test, its conduct or interpretation differ from review questions?	Yes<br>No<br>Unclear
**Reference standard**	Risk of bias	Is the index test compared to a reference standard	Yes<br>No<br>Unclear
		Were the reference standard results interpreted without knowledge of the results of the index test	Yes<br>No<br>Unclear
	Applicability concern	Are there concerns that the target condition as defined by the reference standard does not match the review questions?	Yes<br>No<br>Unclear
**Flow of participants**	Risk of bias	Is the attrition rate acceptable (<20%)?	Yes<br>No<br>Unclear
	Applicability concern	Could the drop out participants or missing data be systematically similar to those who completed?	Yes<br>No<br>Unclear

Appendix B2: QUADAS-2 Scoring guideline (order of answers not important)

	**Risk of bias**	
**Score**	**Question 1**	**Question 2**
**Low**	Yes	Yes
	Yes	Unclear
**High**	No	No
	No	Unclear

**Unclear**	Yes	No
	Unclear	Unclear

**Concern about applicability**	
**Low**	Yes
**High**	No
**Unclear**	Unclear

Appendix C: Quality of individual studies

Study	RISK OF BIAS				CONCERN ABOUT APPLICABILITY		
	Patient selection	Index test	Reference standard	Participants flow	Patient selection	Index test	Reference standard
Singh S et al, 2012	L	L	H	L	L	L	L
Carle C et al, 2013	L	L	L	U	L	L	U
Singh A et al, 2016	L	L	H	L	L	L	U
Hedriana HL et al, 2016	U	L	U	L	L	L	H
Ryan HM et al, 2017	L	L	H	L	L	L	L
Paternino-Caicedo et al, 2017	U	L	L	L	L	L	L
Edwards ES et al, 2015	U	L	L	H	L	L	L
Lappen RJ et al, 2010	U	U	L	H	L	H	U
Von-Dadelszen P et al, 2011	L	L	U	L	L	H	U
Payne BA et al,	L	L	U	L	L	H	U

2014							
Nathan HL et al, 2017	L	U	H	L	L	U	H
Austin DM et al, 2013	H	L	U	L	L	U	U
Maguire PJ et al, Karen AP 2015	L	L	U	U	L	L	U
Maguire PJ, Amy C.O et al, 2015	L	L	U	H	L	L	U
Shields E L et al 2016	L	L	U	H	L	L	L
Sheikh S et al, 2017	L	H	U	L	L	H	U
Merriel A et al, 2017	U	L	U	L	L	L	U

L = Low, H = High, U=Unclear
